# Exchange-correlation kernel for perturbation dependent auxiliary functions in auxiliary density perturbation theory

**DOI:** 10.1007/s00894-024-06091-z

**Published:** 2024-08-08

**Authors:** Luis I. Hernández-Segura, Flor A. Olvera-Rubalcava, Roberto Flores-Moreno, Patrizia Calaminici, Andreas M. Köster

**Affiliations:** 1 Chemistry Department, CINVESTAV, Av. Instituto Politecnico Nacional 2508, Col. San Pedro Zacatenco, Del. Gustavo A. Madero, Mexico City C.P. 07360 Mexico; 2 Departamento de Química, Universidad de Guadalajara, Blvd. Gral. Marcelino García Barragán 1421, Guadalajara, Jal. C.P. 44430 Mexico

**Keywords:** ADFT, Exchange-correlation functional derivatives, deMon2k, LibXC, Analytic frequency calculation, Finite difference exchange-correlation kernel

## Abstract

**Context:**

Analytic exchange-correlation kernel formulations are of the outermost importance for density functional theory (DFT) perturbation calculations. In this paper, the working equation for the exchange-correlation kernel of the generalized gradient approximation (GGA) for perturbation dependent auxiliary functions is derived and discussed in the framework of auxiliary density functional theory (ADFT). The presented new formulation is extended to the unrestricted approach, too. A comprehensive discussion of the implementation of the GGA ADFT kernel, using either the native exchange-correlation functional implementations in deMon2k or the ones from the LibXC library, is given. Calculations with analytic exchange-correlation kernels are compared to their finite difference counterparts. The obtained results are in quantitative agreement. Nevertheless, analytic GGA ADFT kernel implementations show substantial improvement in the computational performance. Similar results are reported for analytic second derivatives of effective core potential (ECP) and model core potential (MCP) matrix elements when compared to their finite difference counterparts in molecular frequency analyses.

**Method:**

All calculations are performed in the framework of ADFT as implemented in deMon2k. In the ADFT analytic frequency calculations, auxiliary density perturbation theory was used. The underlying two-center exchange-correlation kernel matrix elements are calculated by numerical integration either with analytic or finite difference kernel expressions. Validation calculations are performed with the VWN and PBE functionals employing DFT-optimized DZVP basis sets in conjunction with automatically generated GEN-A2 auxiliary density function sets. In the (Pt_3_Cu)_n_ cluster benchmark calculations, the RPBE functional was used. For Pt atoms, the quasi-relativistic LANL2DZ effective core potential with the corresponding valence basis set was employed, whereas for Cu atoms, the all-electron DFT-optimized TZVP basis was applied. The auxiliary density was expanded by the automatically generated GEN-A2* auxiliary function set. We run all benchmark calculations in parallel on 24 cores.

## Introduction

Over the last three decades, Kohn-Sham density functional theory (DFT) [1, 2] methods have become the workhorse in computational chemistry and materials science [3]. Their excellent performance-to-accuracy ratio permits reliable energy and structure predictions for systems with several hundreds of atoms. Although there exists still an accuracy gap to the targeted chemical accuracy of 1 kcal/mol, it is foreseeable that this gap will close with the further development of Kohn-Sham DFT. To this end, it is important to improve density functional approximations (DFAs) such that the overall computational performance is not jeopardized. A possible framework for such developments is auxiliary density functional theory (ADFT) [4] which is based on the variational density fitting for Coulomb [5, 6] and Fock [7, 8] energies and uses the resulting auxiliary density for the evaluation of the exchange-correlation contributions [9, 10]. As a result, ADFT local density approximation (LDA) and generalized gradient approximation (GGA) as well as hybrid calculations have a formal cubic scaling with respect to the number of basis functions. No four-center electron repulsion integral (ERI) evaluations nor basis function product evaluations at grid points are needed in ADFT. Despite this enormous computational simplification, ADFT provides for a given DFA the same accuracy in terms of structure parameters, relative energies, and harmonic frequencies, to name a few, as corresponding Kohn-Sham calculations. Therefore, ADFT is particularly well suited for the reliable optimization of complex nanometric systems like large transition metal clusters [11, 12] or Born-Oppenheimer molecular dynamics simulations [13, 14].

Because the ADFT energy expression is variational, analytic energy derivatives can be straightforwardly calculated, e.g., energy gradients by the 2n+1 theorem of perturbation theory [15]. This permits an alternative perturbation theory formulation to the commonly employed coupled-perturbed Kohn-Sham (CPKS) approach [16]. We have named this alternative approach auxiliary density perturbation theory (ADPT) [17, 18] to emphasize its close connection to ADFT. Since its initial formulation, ADPT has been used to calculate polarizabilities [19–23], Fukui functions [24, 25], electron binding energies [26], alchemical derivatives [27], and various magnetic properties [28–34]. In all cases, CPKS equivalent results were obtained with a much reduced computational demand. In fact, for LDA and GGA perturbation calculations, timings comparable to the ADFT self-consistent field (SCF) approach are observed. More recently, this has been extended to time-dependent ADFT excited state calculations obtaining similar computational performance [35–37]. One reason for the improved computational performance of ADPT arises from the numerical exchange-correlation kernel calculations, i.e., the second functional derivatives of the exchange-correlation energy. In ADPT, these matrix elements contain only auxiliary functions from two centers. As a result, the corresponding kernel matrix has only the dimension of the number of auxiliary functions. Key ingredients for the efficient exchange-correlation kernel calculations are analytic formulas for the various LDA and GGA functionals. Whereas the LDA ADFT kernel formulas are rather straightforward to derive, the corresponding GGA formulas are more involved and deserve some attention. For perturbation-independent auxiliary functions, the generic formulas for closed-shell ADFT kernel calculations have been published a few years ago [38]. However, the recent development of ADFT second analytic energy derivatives [39] employing ADPT requires corresponding kernel calculations for perturbation dependent auxiliary functions. Again, computationally efficient formulations are needed because we aim for frequency analyses of systems with many hundreds to several thousands of atoms. Such calculations are not only mandatory for the characterization of the optimized structures but also for initial Hessian matrix calculations, either for structure minimizations or transition state optimizations. Again, the focus is on GGA kernel formulas which we will derive in this work. To keep the discussion most general, we present the GGA kernel working equations in the framework of the unrestricted formulation. Furthermore, we also validate the recently implemented LibXC interface [40] of deMon2k in the context of these kernel calculations. Because we found in some transition metal cluster frequency analyses a computational bottleneck arising from the finite difference calculations of integrals for effective core potential (ECP) and model core potential (MCP) second derivatives, we also report the corresponding analytic second derivative formulas that were newly implemented into deMon2k [41] in the framework of this study.

The paper is organized as follows. The subsequent theory section contains four subsections. After a brief introduction of second analytic ADFT energy derivatives, the generic working formulas for the GGA potential and kernel calculations for perturbation dependent auxiliary functions are presented. The two following subsections discuss the finite difference GGA ADFT kernel formulations that can be employed if explicit kernel expressions for a GGA functional are missing, and the analytical second derivative formulas for ECP and MCP matrix elements. Section 3 provides the computational details for the validation and benchmark calculations that we present and discuss in the following section. All calculations are performed with the deMon2k code using either the native or LibXC exchange-correlation functional implementations. Finally, in Section 5, the conclusions are summarized.

## Theory

In the linear combination of Gaussian-type orbitals (LCGTO) approximation, neglecting for the sake of simplicity explicit spin dependency, the second analytic ADFT energy derivatives with respect to atomic coordinates $\lambda$ and $\eta$ are given by the following [39]:

1 $$\begin{aligned} E^{(\lambda \eta )} \equiv \frac{\partial ^2 E}{\partial \lambda \partial \eta }= &   \sum _{\mu ,\nu }P_{\mu \nu }^{(\eta )}\left( H_{\mu \nu }^{(\lambda )}+\sum _{\bar{k}}\langle \mu \nu ||\bar{k}\rangle ^{(\lambda )}(x_{\bar{k}}+z_{\bar{k}})\right) \nonumber \\  &   +\sum _{\mu ,\nu }P_{\mu \nu }\left( H_{\mu \nu }^{(\lambda \eta )}+\sum _{\bar{k}}\langle \mu \nu ||\bar{k}\rangle ^{(\lambda \eta )}(x_{\bar{k}}+z_{\bar{k}})\right) \nonumber \\  &   + \sum _{\mu ,\nu }\sum _{\bar{k}}P_{\mu \nu }\langle \mu \nu ||\bar{k}\rangle ^{(\lambda )}\left( x_{\bar{k}}^{(\eta )}+z_{\bar{k}}^{(\eta )}\right) \nonumber \\  &   + \sum _{\bar{k}}x_{\bar{k}}^{(\eta )}\langle \bar{k}^{(\lambda )}|v_{xc}[\tilde{\rho }]\rangle + \sum _{\bar{k}}x_{\bar{k}}\langle \bar{k}^{(\lambda \eta )}|v_{xc}[\tilde{\rho }]\rangle \nonumber \\  &   + \sum _{\bar{k}}x_{\bar{k}}\langle \bar{k}^{(\lambda )}|v_{xc}^{(\eta )}[\tilde{\rho }]\rangle -\sum _{\mu ,\nu }W_{\mu \nu }^{(\eta )}S_{\mu \nu }^{(\lambda )}\nonumber \\  &   -\sum _{\mu ,\nu }W_{\mu \nu }S_{\mu \nu }^{(\lambda \eta )}-\sum _{\bar{k},\bar{l}}G_{\bar{k}\bar{l}}^{(\lambda \eta )}x_{\bar{l}}\left( \frac{1}{2}x_{\bar{k}}+z_{\bar{k}}\right) \nonumber \\  &   -\sum _{\bar{k},\bar{l}}G_{\bar{k}\bar{l}}^{(\lambda )}(x_{\bar{k}}+z_{\bar{k}})x_{\bar{l}}^{(\eta )}-\sum _{\bar{k}, \bar{l}}G_{\bar{k} \bar{l}}^{(\lambda )}z_{\bar{k}}^{(\eta )}x_{\bar{l}} \end{aligned}$$

In Eq. (1), superscripts in parentheses denote derivatives with respect to the corresponding atomic coordinates $\lambda$ and $\eta$. Thus, $E^{(\lambda \eta )}$ corresponds to a Hessian matrix element. The Greek letters $\mu$ and $\nu$ denote (contracted) atomic GTOs, whereas the Latin letters with a bar, $\bar{k}$ and $\bar{l}$, represent primitive atom-centered Hermite Gaussian auxiliary functions. Furthermore, $P_{\mu \nu }$ is an element of the (closed-shell) density matrix, and $H_{\mu \nu }$ is an element of the core matrix, incorporating kinetic, nuclear attraction, and any other external potential contributions, e.g., external electric or magnetic fields. The symbol || in the three-center ERI shorthand notation denotes the Coulomb operator $1/|\textbf{r}_1-\textbf{r}_2|$. It also separates functions from electron 1, in the bra, from those of electron 2, in the ket. The Coulomb and exchange-correlation fitting coefficients are denoted by $x_{\bar{k}}$ and $z_{\bar{k}}$, respectively. The matrix elements $S_{\mu \nu }$, $W_{\mu \nu }$ and $G_{\bar{k}\bar{l}}$ belong to the overlap, energy-weighted density, and Coulomb matrices. Superscripts on matrix elements or fitting coefficients indicate derivatives with respect to corresponding atomic coordinates.

### Analytic GGA exchange-correlation potential

Of particular interest to our further discussion are the matrix elements in Eq. (1) that contain $v_{xc}$ and $v_{xc}^{(\eta )}$, which are the auxiliary density exchange-correlation potential and its derivatives that result in the corresponding kernel expressions. Note that in ADFT, all these expressions are evaluated with the auxiliary density obtained from the variational fitting of Coulomb and Fock energies. In the case of a GGA functional, the exchange-correlation energy takes the following form:

2 $$\begin{aligned} E_{xc}[\tilde{\rho },\tilde{\gamma }] = \int e_{xc}(\tilde{\rho },\tilde{\gamma }) \hspace{3pt} d\textbf{r} \end{aligned}$$

In Eq. (2), $e_{xc}(\tilde{\rho },\tilde{\gamma })$ denotes the density-weighted exchange-correlation energy density depending on the auxiliary density, $\tilde{\rho }(\textbf{r})$, and its gradient square, $\tilde{\gamma }(\textbf{r})$. For simplicity of notation, we omit the explicit position dependencies from the exchange-correlation energy density, potential, and kernel throughout the discussion. In the unrestricted approach, the auxiliary density is expanded as follows:

3 $$\begin{aligned} \tilde{\rho }(\textbf{r}) = \tilde{\rho }^{\alpha }(\textbf{r}) + \tilde{\rho }^{\beta }(\textbf{r}) = \sum _{\bar{k}}x_{\bar{k}}^{\alpha } \bar{k}(\textbf{r}) + \sum _{\bar{k}}x_{\bar{k}}^{\beta } \bar{k}(\textbf{r}) \end{aligned}$$

The $x_{\bar{k}}^\alpha$ and $x_{\bar{k}}^\beta$ are the spin-polarized Coulomb fitting coefficients, obtained from separate spin-dependent fitting equations. The density gradient corrections are included by the scalar $\tilde{\gamma }^{\sigma \tau }(\textbf{r})$ field, with $\sigma$ and $\tau$ being labels for the spin, either $\alpha$ or $\beta$:

4 $$\begin{aligned} \tilde{\gamma }^{\sigma \tau }(\textbf{r}) = \mathbf {\nabla }\tilde{\rho }^\sigma (\textbf{r}) \cdot \mathbf {\nabla }\tilde{\rho }^\tau (\textbf{r}) \end{aligned}$$

Therefore, the explicit form of the unrestricted exchange-correlation energy in Eq. (2) is given by the following:

5 $$\begin{aligned} E_{xc}[\tilde{\rho },\tilde{\gamma }]= &   E_{xc}[\tilde{\rho }^\alpha ,\tilde{\rho }^\beta ,\tilde{\gamma }^{\alpha \alpha },\tilde{\gamma }^{\alpha \beta },\tilde{\gamma }^{\beta \alpha },\tilde{\gamma }^{\beta \beta }]\nonumber \\= &   \int e_{xc}\left( \tilde{\rho }^\alpha ,\tilde{\rho }^\beta ,\tilde{\gamma }^{\alpha \alpha },\tilde{\gamma }^{\alpha \beta },\tilde{\gamma }^{\beta \alpha },\tilde{\gamma }^{\beta \beta }\right) d\textbf{r} \end{aligned}$$

The first derivative of the unrestricted exchange-correlation energy, Eq. (5), with respect to the atomic coordinate $\lambda$ yields the following:

6 $$\begin{aligned} \frac{\partial E_{xc}[\tilde{\rho },\tilde{\gamma }]}{\partial \lambda }= &   \sum _\sigma ^{\alpha , \beta }\int \frac{\partial e_{xc}(\tilde{\rho },\tilde{\gamma })}{\partial \tilde{\rho }^{\sigma }(\textbf{r})} \frac{\partial \tilde{\rho }^{\sigma }(\textbf{r})}{\partial \lambda } \hspace{3pt} d\textbf{r}\nonumber \\  &   + \sum _\sigma ^{\alpha , \beta }\sum _u^{x,y,z}\int \frac{\partial e_{xc}(\tilde{\rho },\tilde{\gamma })}{\partial \tilde{\rho }^\sigma _u(\textbf{r})} \frac{\partial \tilde{\rho }^\sigma _u(\textbf{r})}{\partial \lambda } \hspace{3pt} d\textbf{r} \end{aligned}$$

For the sake of clarity of presentation, we use explicit summation in Eq. (6). The shorthand notation $\tilde{\rho }^\sigma _u(\textbf{r})$ stands for $\partial \tilde{\rho }^{\sigma }/\partial u$ with *u* being *x*, *y*, and *z*. Thus, $\tilde{\rho }^\sigma _u(\textbf{r})$ denotes the components of the auxiliary density gradient, $\mathbf {\nabla }\tilde{\rho }^{\sigma }(\textbf{r})$, with respect to the electronic coordinates. Expanding the auxiliary density and density gradient derivatives as,

7 $$\begin{aligned} \frac{\partial \tilde{\rho }^{\sigma }(\textbf{r})}{\partial \lambda } =&\sum _{\bar{k}}x_{\bar{k}}^{\sigma (\lambda )} \bar{k}(\textbf{r}) + \sum _{\bar{k}}x_{\bar{k}}^{\sigma } \bar{k}^{(\lambda )}(\textbf{r})\end{aligned}$$

8 $$\begin{aligned} \multicolumn{2}{l}{\text{ and }}\\ \frac{\partial \tilde{\rho }^\sigma _u(\textbf{r})}{\partial \lambda } =&\sum _{\bar{k}}x_{\bar{k}}^{\sigma (\lambda )} \frac{\partial \bar{k}(\textbf{r})}{\partial u} + \sum _{\bar{k}}x_{\bar{k}}^{\sigma } \frac{\partial \bar{k}^{(\lambda )}(\textbf{r})}{\partial u}, \end{aligned}$$

yields the following:

9 $$\begin{aligned} \frac{\partial E_{xc}[\tilde{\rho },\tilde{\gamma }]}{\partial \lambda }= &   \sum _\sigma ^{\alpha , \beta }\sum _{\bar{k}}x_{\bar{k}}^{\sigma (\lambda )} \int \left[ \frac{\partial e_{xc}(\tilde{\rho },\tilde{\gamma })}{\partial \tilde{\rho }^{\sigma }(\textbf{r})} + \sum _u^{x,y,z}\frac{\partial e_{xc}(\tilde{\rho },\tilde{\gamma })}{\partial \tilde{\rho }^\sigma _u(\textbf{r})} \hspace{3pt} \frac{\partial }{\partial u} \right] \bar{k}(\textbf{r}) \hspace{3pt} d\textbf{r}\hspace{3pt} \nonumber \\  &   +\sum _\sigma ^{\alpha , \beta }\sum _{\bar{k}}x_{\bar{k}}^{\sigma } \int \left[ \frac{\partial e_{xc}(\tilde{\rho },\tilde{\gamma })}{\partial \tilde{\rho }^{\sigma }(\textbf{r})} + \sum _u^{x,y,z}\frac{\partial e_{xc}(\tilde{\rho },\tilde{\gamma })}{\partial \tilde{\rho }^\sigma _u(\textbf{r})} \hspace{1pt} \frac{\partial }{\partial u}\right] \hspace{1pt} \bar{k}^{(\lambda )}(\textbf{r})\hspace{1pt} d\textbf{r}.\nonumber \\ \end{aligned}$$

Defining the unrestricted GGA ADFT exchange-correlation potential operator [38],

10 $$\begin{aligned} v_{xc}^\sigma [\tilde{\rho },\tilde{\gamma }]\equiv \frac{\partial e_{xc}(\tilde{\rho },\tilde{\gamma })}{\partial \tilde{\rho }^{\sigma }(\textbf{r})} + \sum _u^{x,y,z}\frac{\partial e_{xc}(\tilde{\rho },\tilde{\gamma })}{\partial \tilde{\rho }^\sigma _u(\textbf{r})}\frac{\partial }{\partial u}, \end{aligned}$$

allows the following shorthand notation for the exchange-correlation energy derivative of Eq. (6):

11 $$\begin{aligned} \frac{\partial E_{xc}[\tilde{\rho },\tilde{\gamma }]}{\partial \lambda }= &   \sum _\sigma ^{\alpha , \beta }\sum _{\bar{k}}x_{\bar{k}}^{\sigma (\lambda )}\left\langle \bar{k}|v_{xc}^\sigma [\tilde{\rho },\tilde{\gamma }]\right\rangle \nonumber \\  &   + \sum _\sigma ^{\alpha , \beta }\sum _{\bar{k}}x_{\bar{k}}^{\sigma }\left\langle \bar{k}^{(\lambda )}\Big |v_{xc}^\sigma [\tilde{\rho },\tilde{\gamma }]\right\rangle \end{aligned}$$

Note that the first term of Eq. (11) will be absorbed in the Pulay term of the ADFT energy gradients [42] and, therefore, will not be explicitly calculated. Terms analogous to the second sum of Eq. (11) appear in the Hessian matrix elements given in Eq. (1) in the form of the $\left\langle {\bar{k}}^{(\lambda )}|{v_{xc}[\tilde{\rho }]}\right\rangle$ and $\left\langle {\bar{k}}^{(\lambda \eta )}|{v_{xc}[\tilde{\rho }]}\right\rangle$ matrix elements. Whereas the first terms are explicitly evaluated in the response ADPT calculations, the second term’s contributions to the skeleton Hessian matrix are indirectly evaluated by translational invariance. This permits the use of moderate grids for the numerical integration in the Hessian matrix calculation even without the explicit inclusion of atomic weight function derivatives [43].

For the implementation in deMon2k, it is more convenient to formulate the exchange-correlation potential of Eq. (10) in terms of $\tilde{\rho }^{\sigma }(\textbf{r})$ and $\tilde{\gamma }^{\sigma \tau }(\textbf{r})$. To this end, we apply the chain rule to Eq. (10) obtaining the following:

12 $$\begin{aligned} v_{xc}^{\sigma }[\tilde{\rho },\tilde{\gamma }] \equiv \frac{\partial e_{xc}(\tilde{\rho },\tilde{\gamma })}{\partial \tilde{\rho }^{\sigma }(\textbf{r})}+\sum _{\sigma ',\tau '}^{\alpha , \beta }\sum _u^{x,y,z}\frac{\partial e_{xc}(\tilde{\rho },\tilde{\gamma })}{\partial \tilde{\gamma }^{\sigma ' \tau '}(\textbf{r})}\frac{\partial \tilde{\gamma }^{\sigma ' \tau '}(\textbf{r})}{\partial \tilde{\rho }^\sigma _u(\textbf{r})}\frac{\partial }{\partial u} \end{aligned}$$

For the derivative of $\tilde{\gamma }^{\sigma ' \tau '}(\textbf{r})$ with respect to $\tilde{\rho }^\sigma _u(\textbf{r})$ holds:

13 $$\begin{aligned} \frac{\partial \tilde{\gamma }^{\sigma ' \tau '}(\textbf{r})}{\partial \tilde{\rho }^\sigma _u(\textbf{r})} = \tilde{\rho }_u^{\sigma '}(\textbf{r})\delta _{\sigma \tau '} + \tilde{\rho }_u^{\tau '}(\textbf{r})\delta _{\sigma \sigma '} \end{aligned}$$

Inserting Eq. (13) into Eq. (12) and the result into Eq. (11) yields the explicit form of the unrestricted exchange-correlation energy derivative as implemented in deMon2k:

14 $$\begin{aligned} \frac{\partial E_{xc}[\tilde{\rho },\tilde{\gamma }]}{\partial \lambda }= &   \sum _\sigma ^{\alpha , \beta }\sum _{\bar{k}}x_{\bar{k}}^{\sigma (\lambda )} \int \Bigg [ \frac{\partial e_{xc}(\tilde{\rho },\tilde{\gamma })}{\partial \tilde{\rho }^{\sigma }(\textbf{r})} \nonumber \\  &   + 2\sum _{\sigma '}^{\alpha ,\beta }\sum _u^{x,y,z}\frac{\partial e_{xc}(\tilde{\rho },\tilde{\gamma })}{\partial \tilde{\gamma }^{\sigma \sigma '} (\textbf{r})} \tilde{\rho }_u^{\sigma '}(\textbf{r})\frac{\partial }{\partial u}\Bigg ] \bar{k}(\textbf{r})\hspace{3pt} d\textbf{r}\hspace{3pt} \nonumber \\  &   + \sum _\sigma ^{\alpha , \beta }\sum _{\bar{k}}x_{\bar{k}}^{\sigma } \int \Bigg [ \frac{\partial e_{xc}(\tilde{\rho },\tilde{\gamma })}{\partial \tilde{\rho }^{\sigma }(\textbf{r})} \nonumber \\  &   + 2 \sum _{\sigma '}^{\alpha ,\beta }\sum _u^{x,y,z}\frac{\partial e_{xc}(\tilde{\rho },\tilde{\gamma })}{\partial \tilde{\gamma }^{\sigma \sigma '}(\textbf{r})}\tilde{\rho }_u^{\sigma '}(\textbf{r})\frac{\partial }{\partial u}\Bigg ] \bar{k}^{(\lambda )}(\textbf{r})\hspace{3pt} d\textbf{r}\nonumber \\ \end{aligned}$$

The corresponding exchange-correlation potential, $v_{xc}^{\sigma }[\tilde{\rho },\tilde{\gamma }]$, is given by the following:

15 $$\begin{aligned} v_{xc}^{\sigma }[\tilde{\rho },\tilde{\gamma }] \equiv \frac{\partial e_{xc}(\tilde{\rho },\tilde{\gamma })}{\partial \tilde{\rho }^{\sigma }(\textbf{r})} + 2\sum _{\sigma '}^{\alpha ,\beta }\sum _u^{x,y,z}\frac{\partial e_{xc}(\tilde{\rho },\tilde{\gamma })}{\partial \tilde{\gamma }^{\sigma \sigma '}(\textbf{r})}\tilde{\rho }_u^{\sigma '}(\textbf{r})\frac{\partial }{\partial u}\end{aligned}$$

### Analytic GGA exchange-correlation kernel

We now turn to the analytic GGA ADFT exchange-correlation kernel calculation in ADPT. To this end, we derive Eq. (11) with respect to a second atomic coordinate keeping in mind that the first term of Eq. (11) is absorbed by the Pulay relation. Thus, only the second sum of Eq. (11) needs to be explicitly considered:

16 $$\begin{aligned} \frac{\partial ^2 E_{xc}[\tilde{\rho },\tilde{\gamma }]}{\partial \eta \partial \lambda }\Bigg |_{\text {explicit}}= &   \frac{\partial }{\partial \eta }\sum _\sigma ^{\alpha , \beta }\sum _{\bar{k}}x_{\bar{k}}^{\sigma }\left\langle \bar{k}^{(\lambda )}\Big |v_{xc}^\sigma [\tilde{\rho },\tilde{\gamma }]\right\rangle \nonumber \\= &   \sum _\sigma ^{\alpha , \beta }\sum _{\bar{k}}x_{\bar{k}}^{\sigma (\eta )}\left\langle \bar{k}^{(\lambda )}\Big |v_{xc}^\sigma [\tilde{\rho },\tilde{\gamma }]\right\rangle \nonumber \\  &   +\sum _\sigma ^{\alpha , \beta }\sum _{\bar{k}}x_{\bar{k}}^{\sigma }\left\langle \bar{k}^{(\lambda \eta )}\Big |v_{xc}^\sigma [\tilde{\rho },\tilde{\gamma }]\right\rangle \nonumber \\  &   +\sum _\sigma ^{\alpha , \beta }\sum _{\bar{k}}x_{\bar{k}}^{\sigma } \left\langle \bar{k}^{(\lambda )}\Bigg |\frac{\partial v_{xc}^\sigma [\tilde{\rho },\tilde{\gamma }]}{\partial \eta } \right\rangle \nonumber \\ \end{aligned}$$

The first two terms of Eq. (16) are already discussed in Section 2.1 and can be calculated by the exchange-correlation potential expressions given above. Thus, we focus in the following only on the third term of Eq. (16).

To proceed, we derive the exchange-correlation potential with respect to the atomic coordinate $\eta$:

17 $$\begin{aligned} \frac{\partial v_{xc}^\sigma [\tilde{\rho },\tilde{\gamma }]}{\partial \eta }= &   \sum _\tau ^{\alpha , \beta }\int \frac{\partial v_{xc}^{\sigma }(\tilde{\rho },\tilde{\gamma })}{\partial \tilde{\rho }^{\tau }(\textbf{r}')} \frac{\partial \tilde{\rho }^{\tau }(\textbf{r}')}{\partial \eta }\hspace{3pt} d\textbf{r}'\nonumber \\  &   + \sum _\tau ^{\alpha , \beta }\sum _v^{x,y,z}\int \frac{\partial v_{xc}^{\sigma }(\tilde{\rho },\tilde{\gamma })}{\partial \tilde{\rho }^\tau _v(\textbf{r}')} \frac{\partial \tilde{\rho }^\tau _v(\textbf{r}')}{\partial \eta }\hspace{3pt} d\textbf{r}'\end{aligned}$$

Expanding the auxiliary density and density gradient derivatives according to Eqs. (7) and (8) results into the following:

18 $$\begin{aligned} \frac{\partial v_{xc}^\sigma [\tilde{\rho },\tilde{\gamma }]}{\partial \eta }= &   \sum _\tau ^{\alpha , \beta }\sum _{\bar{l}}x_{\bar{l}}^{\tau (\eta )} \Bigg [\int \frac{\partial v_{xc}^{\sigma }(\tilde{\rho },\tilde{\gamma })}{\partial \tilde{\rho }^{\tau }(\textbf{r}')}\hspace{3pt}\bar{l}(\textbf{r}')\hspace{3pt} d\textbf{r}'\nonumber \\  &   + \sum _v^{x,y,z}\int \frac{\partial v_{xc}^{\sigma }(\tilde{\rho },\tilde{\gamma })}{\partial \tilde{\rho }^\tau _v(\textbf{r}')}\hspace{3pt}\frac{\partial \bar{l}(\textbf{r}')}{\partial v'}\hspace{3pt} d\textbf{r}'\Bigg ] \nonumber \\  &   + \sum _\tau ^{\alpha , \beta }\sum _{\bar{l}}x_{\bar{l}}^{\tau } \Bigg [\int \frac{\partial v_{xc}^{\sigma }(\tilde{\rho },\tilde{\gamma })}{\partial \tilde{\rho }^{\tau }(\textbf{r}')}\hspace{3pt}\bar{l}^{(\eta )}(\textbf{r}')\hspace{3pt} d\textbf{r}'\nonumber \\  &   + \sum _v^{x,y,z}\int \frac{\partial v_{xc}^{\sigma }(\tilde{\rho },\tilde{\gamma })}{\partial \tilde{\rho }^\tau _v(\textbf{r}')}\hspace{3pt}\frac{\partial \bar{l}^{(\eta )}(\textbf{r}')}{\partial v'}\hspace{3pt} d\textbf{r}'\Bigg ] \end{aligned}$$

Inserting the explicit expression of the GGA exchange-correlation potential from Eq. (10) into Eq. (18) yields the following:

19 $$\begin{aligned} \frac{\partial v_{xc}^\sigma [\tilde{\rho },\tilde{\gamma }]}{\partial \eta }= &   \sum _\tau ^{\alpha , \beta }\sum _{\bar{l}}x_{\bar{l}}^{\tau (\eta )} \Bigg [\int \frac{\partial ^2 e_{xc}(\tilde{\rho },\tilde{\gamma })}{\partial \tilde{\rho }^{\tau }(\textbf{r}') \partial \tilde{\rho }^{\sigma }(\textbf{r})}\hspace{3pt}\bar{l}(\textbf{r}')\hspace{3pt} d\textbf{r}'\nonumber \\  &   + \sum _u^{x,y,z}\int \frac{\partial ^2 e_{xc}(\tilde{\rho },\tilde{\gamma })}{\partial \tilde{\rho }^{\tau }(\textbf{r}') \partial \tilde{\rho }^\sigma _u(\textbf{r})}\hspace{3pt}\bar{l}(\textbf{r}')\hspace{3pt} d\textbf{r}'\hspace{3pt} \frac{\partial }{\partial u}\Bigg ] \nonumber \\  &   + \sum _\tau ^{\alpha , \beta }\sum _{\bar{l}}x_{\bar{l}}^{\tau (\eta )} \Bigg [\sum _v^{x,y,z}\int \frac{\partial ^2 e_{xc}(\tilde{\rho },\tilde{\gamma })}{\partial \tilde{\rho }^\tau _v(\textbf{r}') \partial \tilde{\rho }^{\sigma }(\textbf{r})}\hspace{3pt}\frac{\partial \bar{l}(\textbf{r}')}{\partial v'}\hspace{3pt} d\textbf{r}'\nonumber \\  &   + \sum _{u,v}^{x,y,z}\int \frac{\partial ^2 e_{xc}(\tilde{\rho },\tilde{\gamma })}{\partial \tilde{\rho }^\tau _v(\textbf{r}') \partial \tilde{\rho }^\sigma _u(\textbf{r})}\hspace{3pt}\frac{\partial \bar{l}(\textbf{r}')}{\partial v'}\hspace{3pt} d\textbf{r}'\hspace{3pt} \frac{\partial }{\partial u}\Bigg ] \nonumber \\  &   + \sum _\tau ^{\alpha , \beta }\sum _{\bar{l}}x_{\bar{l}}^{\tau } \Bigg [\int \frac{\partial ^2 e_{xc}(\tilde{\rho },\tilde{\gamma })}{\partial \tilde{\rho }^{\tau }(\textbf{r}') \partial \tilde{\rho }^{\sigma }(\textbf{r})}\hspace{3pt}\bar{l}^{(\eta )}(\textbf{r}')\hspace{3pt} d\textbf{r}'\nonumber \\  &   + \sum _u^{x,y,z}\int \frac{\partial ^2 e_{xc}(\tilde{\rho },\tilde{\gamma })}{\partial \tilde{\rho }^{\tau }(\textbf{r}') \partial \tilde{\rho }^\sigma _u(\textbf{r})}\hspace{3pt}\bar{l}^{(\eta )}(\textbf{r}')\hspace{3pt} d\textbf{r}'\hspace{3pt} \frac{\partial }{\partial u}\Bigg ] \nonumber \\  &   + \sum _\tau ^{\alpha , \beta }\sum _{\bar{l}}x_{\bar{l}}^{\tau } \Bigg [\sum _v^{x,y,z}\int \frac{\partial ^2 e_{xc}(\tilde{\rho },\tilde{\gamma })}{\partial \tilde{\rho }^\tau _v(\textbf{r}') \partial \tilde{\rho }^{\sigma }(\textbf{r})}\hspace{3pt}\frac{\partial \bar{l}^{(\eta )}(\textbf{r}')}{\partial v'}\hspace{3pt} d\textbf{r}'\nonumber \\  &   + \sum _{u,v}^{x,y,z}\int \frac{\partial ^2 e_{xc}(\tilde{\rho },\tilde{\gamma })}{\partial \tilde{\rho }^\tau _v(\textbf{r}') \partial \tilde{\rho }^\sigma _u(\textbf{r})}\hspace{3pt}\frac{\partial \bar{l}^{(\eta )}(\textbf{r}')}{\partial v'}\hspace{3pt} d\textbf{r}'\hspace{3pt} \frac{\partial }{\partial u}\Bigg ] \end{aligned}$$

At this point, it is convenient to define the GGA ADFT exchange-correlation kernel according to Eq. (19). Therefore, we obtain as GGA kernel expression in ADFT:

20 $$\begin{aligned} f_{xc}^{\sigma \tau }(\tilde{\rho },\tilde{\gamma })\equiv &   \hspace{3pt} \frac{\partial ^2 e_{xc}(\tilde{\rho },\tilde{\gamma })}{\partial \tilde{\rho }^{\tau }(\textbf{r}') \partial \tilde{\rho }^{\sigma }(\textbf{r})} + \sum _u^{x,y,z}\frac{\partial ^2 e_{xc}(\tilde{\rho },\tilde{\gamma })}{\partial \tilde{\rho }^{\tau }(\textbf{r}') \partial \tilde{\rho }^\sigma _u(\textbf{r})} \frac{\partial }{\partial u} \hspace{3pt} \nonumber \\  &   + \hspace{3pt} \sum _v^{x,y,z}\frac{\partial ^2 e_{xc}(\tilde{\rho },\tilde{\gamma })}{\partial \tilde{\rho }^\tau _v(\textbf{r}') \partial \tilde{\rho }^{\sigma }(\textbf{r})}\frac{\partial }{\partial v'}\nonumber \\  &   + \sum _{u,v}^{x,y,z}\frac{\partial ^2 e_{xc}(\tilde{\rho },\tilde{\gamma })}{\partial \tilde{\rho }^\tau _v(\textbf{r}') \partial \tilde{\rho }^\sigma _u(\textbf{r})}\frac{\partial }{\partial v'}\frac{\partial }{\partial u} \end{aligned}$$

As Eq. (20) shows, the exchange-correlation kernel has in general four spin contributions, namely $\alpha \alpha , \alpha \beta , \beta \alpha$, and $\beta \beta$. As developed in Eq. (20), the kernel is also non-local, i.e., depends from $\textbf{r}$ and $\textbf{r}'$. However, for LDA and GGA, $e_{xc}(\tilde{\rho },\tilde{\gamma })$ is an ordinary function. As a result, the variables $\textbf{r}$ and $\textbf{r}'$ collapse in the integration over the exchange-correlation kernel as outlined in [38] and [44].

Back-substitution of the kernel definition, Eq. (20), into the exchange-correlation potential derivative, Eq. (19), yields for the matrix element in the last term of Eq. (16):

21 $$\begin{aligned}  &   \left\langle \bar{k}^{(\lambda )}\Bigg |\frac{\partial v_{xc}^\sigma [\tilde{\rho },\tilde{\gamma }]}{\partial \eta } \right\rangle \nonumber \\= &   \sum _\tau ^{\alpha , \beta }\sum _{\bar{l}}x_{\bar{l}}^{\tau (\eta )} \left[ \iint f_{xc}^{\sigma \tau } (\tilde{\rho },\tilde{\gamma })\hspace{3pt} \bar{l}(\textbf{r}') \hspace{3pt} \bar{k}^{(\lambda )}(\textbf{r})\hspace{3pt} \delta (\textbf{r}-\textbf{r}')\hspace{3pt} d\textbf{r}\hspace{3pt} d\textbf{r}'\right] \nonumber \\  &   +\sum _\tau ^{\alpha , \beta }\sum _{\bar{l}}x_{\bar{l}}^{\tau } \left[ \iint f_{xc}^{\sigma \tau } (\tilde{\rho },\tilde{\gamma })\hspace{1pt} \bar{l}^{(\eta )}(\textbf{r}') \hspace{1pt} \bar{k}^{(\lambda )}(\textbf{r})\hspace{1pt} \delta (\textbf{r}-\textbf{r}')\hspace{1pt} d\textbf{r}\hspace{1pt} d\textbf{r}'\right] \nonumber \\= &   \sum _\tau ^{\alpha , \beta }\sum _{\bar{l}}x_{\bar{l}}^{\tau (\eta )} \left\langle \bar{k}^{(\lambda )}\Big |f_{xc}^{\sigma \tau }[\tilde{\rho },\tilde{\gamma }]\Big |\bar{l}\right\rangle \nonumber \\  &   + \sum _\tau ^{\alpha , \beta }\sum _{\bar{l}}x_{\bar{l}}^{\tau } \left\langle \bar{k}^{(\lambda )}\Big |f_{xc}^{\sigma \tau }[\tilde{\rho },\tilde{\gamma }]\Big |\bar{l}^{(\eta )}\right\rangle \end{aligned}$$

Therefore, we find explicit contributions to the ADFT Hessian matrix elements from the second derivatives of the exchange-correlation energy functional with respect to the atomic coordinates $\eta$ and $\lambda$:

22 $$\begin{aligned} \frac{\partial ^2 E_{xc}[\tilde{\rho },\tilde{\gamma }]}{\partial \eta \partial \lambda }\Bigg |_{\text {explicit}}= &   \sum _\sigma ^{\alpha , \beta }\sum _{\bar{k}}x_{\bar{k}}^{\sigma (\eta )} \left\langle \bar{k}^{(\lambda )}|v_{xc}^\sigma [\tilde{\rho },\tilde{\gamma }]\right\rangle \nonumber \\  &   + \sum _\sigma ^{\alpha , \beta }\sum _{\bar{k}}x_{\bar{k}}^\sigma \left\langle \bar{k}^{(\eta \lambda )}|v_{xc}^\sigma [\tilde{\rho },\tilde{\gamma }]\right\rangle \nonumber \\  &   + \sum _{\sigma ,\tau }^{\alpha , \beta }\sum _{\bar{k},\bar{l}}x_{\bar{k}}^\sigma \left\langle \bar{k}^{(\lambda )}\Big |f_{xc}^{\sigma \tau }[\tilde{\rho },\tilde{\gamma }]\Big |\bar{l}\right\rangle x_{\bar{l}}^{\tau (\eta )} \nonumber \\  &   + \sum _{\sigma ,\tau }^{\alpha , \beta }\sum _{\bar{k},\bar{l}}x_{\bar{k}}^\sigma \left\langle \bar{k}^{(\lambda )}\Big |f_{xc}^{\sigma \tau }[\tilde{\rho },\tilde{\gamma }]\Big |\bar{l}^{(\eta )}\right\rangle x_{\bar{l}}^{\tau }\nonumber \\ \end{aligned}$$

For the kernel implementation in deMon2k, it is more convenient to formulate the exchange-correlation kernel of Eq. (20) in terms of $\tilde{\rho }^{\sigma }(\textbf{r})$ and $\tilde{\gamma }^{\sigma \tau }(\textbf{r})$. Applying the chain rule to the terms involving the $\sigma$ and $\tau$ density gradients in Eq. (19) and using Eq. (13), we obtain the following:

23 $$\begin{aligned} \frac{\partial v_{xc}^\sigma [\tilde{\rho },\tilde{\gamma }]}{\partial \eta }= &   \sum _\tau ^{\alpha , \beta }\sum _{\bar{l}}x_{\bar{l}}^{\tau (\eta )} \int \frac{\partial ^2 e_{xc}(\tilde{\rho },\tilde{\gamma })}{\partial \tilde{\rho }^{\tau }(\textbf{r}') \partial \tilde{\rho }^{\sigma }(\textbf{r})}\hspace{3pt}\bar{l}(\textbf{r}')\hspace{3pt} d\textbf{r}'\nonumber \\  &   +2\sum _{\sigma ',\tau }^{\alpha ,\beta }\sum _{\bar{l}}\sum _u^{x,y,z}x_{\bar{l}}^{\tau (\eta )} \int \frac{\partial ^2 e_{xc}(\tilde{\rho },\tilde{\gamma })}{\partial \tilde{\rho }^{\tau }(\textbf{r}') \partial \tilde{\gamma }^{\sigma \sigma '}(\textbf{r})}\hspace{1pt} \bar{l}(\textbf{r}')\hspace{1pt} d\textbf{r}'\hspace{1pt} \tilde{\rho }_u^{\sigma '}(\textbf{r})\frac{\partial }{\partial u}\nonumber \\  &   + 2 \sum _{\tau ,\tau '}^{\alpha ,\beta }\sum _{\bar{l}}\sum _v^{x,y,z}x_{\bar{l}}^{\tau (\eta )} \int \frac{\partial ^2 e_{xc}(\tilde{\rho },\tilde{\gamma })}{\partial \tilde{\gamma }^{\tau \tau '}(\textbf{r}') \partial \tilde{\rho }^{\sigma }(\textbf{r})}\hspace{1pt}\tilde{\rho }_v^{\tau '}(\textbf{r}')\hspace{1pt}\frac{\partial \bar{l}(\textbf{r}')}{\partial v'}\hspace{1pt} d\textbf{r}'\nonumber \\  &   + 4 \sum _{\sigma ',\tau ,\tau '}^{\alpha ,\beta }\sum _{\bar{l}}\sum _{u,v}^{x,y,z}x_{\bar{l}}^{\tau (\eta )} \int \frac{\partial ^2 e_{xc}(\tilde{\rho },\tilde{\gamma })}{\partial \tilde{\gamma }^{\tau \tau '}(\textbf{r}') \partial \tilde{\gamma }^{\sigma \sigma '}(\textbf{r})}\hspace{1pt} \tilde{\rho }_v^{\tau '}(\textbf{r}')\frac{\partial \bar{l}(\textbf{r}')}{\partial v'}\hspace{1pt} d\textbf{r}'\hspace{1pt} \tilde{\rho }_u^{\sigma '}(\textbf{r})\frac{\partial }{\partial u}\nonumber \\  &   + 2 \sum _\tau ^{\alpha , \beta }\sum _{\bar{l}}\sum _v^{x,y,z}x_{\bar{l}}^{\tau (\eta )} \int \frac{\partial e_{xc}(\tilde{\rho },\tilde{\gamma })}{\partial \tilde{\gamma }^{\sigma \tau }(\textbf{r})}\hspace{1pt} \frac{\partial \bar{l}(\textbf{r}')}{\partial v'}\hspace{1pt} d\textbf{r}'\hspace{1pt}\frac{\partial }{\partial v} \nonumber \\  &   + \sum _\tau ^{\alpha , \beta }\sum _{\bar{l}}x_{\bar{l}}^{\tau } \int \frac{\partial ^2 e_{xc}(\tilde{\rho },\tilde{\gamma })}{\partial \tilde{\rho }^{\tau }(\textbf{r}') \partial \tilde{\rho }^{\sigma }(\textbf{r})}\hspace{1pt}\bar{l}^{(\eta )}(\textbf{r}')\hspace{1pt} d\textbf{r}'\nonumber \\  &   + 2 \sum _{\sigma ',\tau }^{\alpha ,\beta }\sum _{\bar{l}}\sum _u^{x,y,z}x_{\bar{l}}^{\tau } \int \frac{\partial ^2 e_{xc}(\tilde{\rho },\tilde{\gamma })}{\partial \tilde{\rho }^{\tau }(\textbf{r}') \partial \tilde{\gamma }^{\sigma \sigma '}(\textbf{r})} \hspace{1pt}\bar{l}^{(\eta )}(\textbf{r}')\hspace{1pt} d\textbf{r}'\hspace{1pt} \tilde{\rho }_u^{\sigma '}(\textbf{r})\frac{\partial }{\partial u}\nonumber \\  &   + 2 \sum _{\tau ,\tau '}^{\alpha ,\beta }\sum _{\bar{l}}\sum _v^{x,y,z}x_{\bar{l}}^{\tau } \int \frac{\partial ^2 e_{xc}(\tilde{\rho },\tilde{\gamma })}{\partial \tilde{\gamma }^{\tau \tau '}(\textbf{r}') \partial \tilde{\rho }^{\sigma }(\textbf{r})}\hspace{1pt}\tilde{\rho }_v^{\tau '}(\textbf{r}')\hspace{1pt}\frac{\partial \bar{l}^{(\eta )}(\textbf{r}')}{\partial v'}\hspace{1pt} d\textbf{r}'\nonumber \\  &   + 4 \sum _{\sigma ',\tau ,\tau '}^{\alpha ,\beta }\sum _{\bar{l}}\sum _{u,v}^{x,y,z}x_{\bar{l}}^{\tau } \int \frac{\partial ^2 e_{xc}(\tilde{\rho },\tilde{\gamma })}{\partial \tilde{\gamma }^{\tau \tau '}(\textbf{r}') \partial \tilde{\gamma }^{\sigma \sigma '}(\textbf{r})}\hspace{1pt}\tilde{\rho }_v^{\tau '}(\textbf{r}')\hspace{1pt}\frac{\partial \bar{l}^{(\eta )}(\textbf{r}')}{\partial v'}\hspace{1pt} d\textbf{r}'\hspace{1pt}\tilde{\rho }_u^{\sigma '}(\textbf{r})\hspace{1pt} \frac{\partial }{\partial u}\nonumber \\  &   + 2 \sum _\tau ^{\alpha , \beta }\sum _{\bar{l}}\sum _v^{x,y,z}x_{\bar{l}}^{\tau } \int \frac{\partial e_{xc}(\tilde{\rho },\tilde{\gamma })}{\partial \tilde{\gamma }^{\sigma \tau }(\textbf{r})} \hspace{1pt}\frac{\partial \bar{l}^{(\eta )}(\textbf{r}')}{\partial v'}\hspace{1pt} d\textbf{r}'\hspace{1pt} \frac{\partial }{\partial v} \end{aligned}$$

Hence, the implemented GGA kernel formulation in ADFT is given by the following:

24 $$\begin{aligned} f_{xc}^{\sigma \tau }(\tilde{\rho },\tilde{\gamma })\equiv &   \hspace{3pt} \frac{\partial ^2 e_{xc}(\tilde{\rho },\tilde{\gamma })}{\partial \tilde{\rho }^{\tau }(\textbf{r}') \partial \tilde{\rho }^{\sigma }(\textbf{r})} \nonumber \\  &   + 2\sum _{\sigma '}^{\alpha ,\beta }\sum _u^{x,y,z}\frac{\partial ^2 e_{xc}(\tilde{\rho },\tilde{\gamma })}{\partial \tilde{\rho }^{\tau }(\textbf{r}') \partial \tilde{\gamma }^{\sigma \sigma '}(\textbf{r})} \tilde{\rho }_u^{\sigma '}(\textbf{r})\frac{\partial }{\partial u} \hspace{3pt} \nonumber \\  &   + 2\sum _{\tau '}^{\alpha , \beta }\sum _v^{x,y,z}\frac{\partial ^2 e_{xc}(\tilde{\rho },\tilde{\gamma })}{\partial \tilde{\gamma }^{\tau \tau '}(\textbf{r}') \partial \tilde{\rho }^{\sigma }(\textbf{r})}\tilde{\rho }^\tau _v(\textbf{r}')\frac{\partial }{\partial v'}\nonumber \\  &   +4 \sum _{\sigma ',\tau '}^{\alpha , \beta }\sum _{u,v}^{x,y,z}\frac{\partial ^2 e_{xc}(\tilde{\rho },\tilde{\gamma })}{\partial \tilde{\gamma }^{\tau \tau '}(\textbf{r}') \partial \tilde{\gamma }^{\sigma \sigma '}(\textbf{r})}\tilde{\rho }^{\tau '}_v(\textbf{r}')\tilde{\rho }^{\sigma '}_u(\textbf{r})\nonumber \\  &   \times \frac{\partial }{\partial v'}\frac{\partial }{\partial u} + 2 \sum _v^{x,y,z}\frac{\partial e_{xc}(\tilde{\rho },\tilde{\gamma })}{\partial \tilde{\gamma }^{\sigma \tau }(\textbf{r})}\frac{\partial }{\partial v'}\frac{\partial }{\partial v} \end{aligned}$$

### Finite difference GGA exchange-correlation kernel

In the last section, the working formula for the unrestricted GGA ADFT kernel, Eq. (24), in deMon2k was presented. This implementation requires second derivatives of the density-weighted exchange-correlation energy density. In cases where these derivatives are not available, the matrix elements of the GGA ADFT kernel can be calculated by a finite difference formulation. This approach requires only the implementation of the corresponding GGA exchange-correlation potential formulas that are mandatory for SCF calculations. To this end, the exchange-correlation potential is evaluated at symmetric arbitrarily small changes in the auxiliary density at a given grid point. Following the definition of derivatives, the unrestricted finite difference formulation of the GGA ADFT kernel can be expressed as follows:

25 $$\begin{aligned} \left\langle \bar{k}^{(\lambda )}\Big | f_{xc}^{\sigma \tau } \Big |\right.= &   \int \frac{\delta v_{xc}^\sigma [\tilde{\rho }^{\tau }(\textbf{r})]}{\delta \tilde{\rho }^\tau (\textbf{r}')}\bar{k}^{(\lambda )}(\textbf{r}')d\textbf{r}'\nonumber \\\approx &   \frac{v_{xc}^\sigma [\tilde{\rho }^{\tau }(\textbf{r})+ \epsilon \bar{k}^{(\lambda )}(\textbf{r})]- v_{xc}^\sigma [\tilde{\rho }^{\tau }(\textbf{r})- \epsilon \bar{k}^{(\lambda )}(\textbf{r})]}{2\epsilon }\nonumber \\ \end{aligned}$$

Equation (25) represents the arbitrary changes in the auxiliary density by $\pm \epsilon \bar{k}^{(\lambda )}(\textbf{r})$, where $\epsilon$ is the step size, and $\bar{k}^{(\lambda )}(\textbf{r})$ is the derivative of the auxiliary function $\bar{k}$ with respect to the atomic coordinate $\lambda$. This choice is made with the intention of constructing the kernel integrals appearing in Eq. (22).

Take as an example the kernel integral in the third term of Eq. (22). It can be expressed by the following finite difference formulation:

26 $$\begin{aligned}  &   \left\langle \bar{k}^{(\lambda )}|f_{xc}^{\tau \sigma }|\bar{l}\right\rangle \\\approx &   \int \frac{v_{xc}^\sigma [\tilde{\rho }^{\tau }(\textbf{r})+ \epsilon \bar{k}^{(\lambda )}(\textbf{r})]- v_{xc}^\sigma [\tilde{\rho }^{\tau }(\textbf{r})- \epsilon \bar{k}^{(\lambda )}(\textbf{r})]}{2\epsilon } \hspace{3pt} \bar{l}(\textbf{r}) \hspace{3pt} d\textbf{r}\nonumber \end{aligned}$$

Equation (26) can be used to calculate the exchange-correlation kernel integrals appearing in Eq. (22) for any GGA functional for which exchange-correlation potential formulas are implemented. The integral in Eq. (26) is evaluated numerically with the same grid used in the SCF. In deMon2k, the step size $\epsilon$ is set to 10^-9^ a.u., which yields consistent and robust results [20].

### Analytic second derivatives of ECPs and MCPs

In the original frequency analysis module of deMon2k, the second derivatives of ECPs and MCPs are calculated by finite differences from the corresponding analytic gradients. For systems with many ECP or MCP centers, these finite difference calculations can become a computational bottleneck. Therefore, we implemented within this work analytic second derivatives for ECPs and MCPs. The relevant contributions to the Hessian matrix from second derivatives of ECPs or MCPs, *U*, centered on atom C have the general form:

27 $$\begin{aligned} H_{C}^{\lambda \eta } = \sum _{\mu \nu } P_{\mu \nu } \frac{\partial ^2}{\partial \lambda \partial \eta } \langle \mu |U|\nu \rangle \end{aligned}$$

Taking $\textbf{r}_C = \textbf{r} -\textbf{C}$ for ECPs, the pseudopotential operator is given by the following:

28 $$\begin{aligned} U_{ECP}(\textbf{r}_C) = U_L(r_C) + \sum _{l=0}^{L-1}\sum _{m=-l}^{l} |S_{lm}(\hat{\textbf{r}}_C)\rangle U_l(r_C) \langle S_{lm}(\hat{\textbf{r}}_C)|. \end{aligned}$$

The first term on the right-hand side is a purely local central force potential located on atom *C*. The second term is a double sum of semi-local operators with a fully local radial part and non-local angular part, here expressed by normalized real spherical harmonics, $S_{lm}(\hat{\textbf{r}}_C)$. In this notation, $\hat{\textbf{r}}_C$ is the unit vector in the direction of $\textbf{r}_C$. For MCPs, a similar operator form is used:

29 $$\begin{aligned} U_{MCP}(\textbf{r}_C) = U_L(r_C) + \sum _{l=0}^{L-1}\sum _{m=-l}^{l} |\phi _{lm}(\textbf{r}_C)\rangle B_l \langle \phi _{lm}(\textbf{r}_C)|. \end{aligned}$$

In Eq. (29), $B_l$ is a constant and $\phi _{lm}(\textbf{r}_C)$ is a core orbital. Since core orbitals are normalized, the terms inside the sum are true projectors, fully non-local. Analytical calculation of second derivatives was implemented taking advantage of translational invariance. Differentiation of the operators, especially the semi-local ECP operator, is not advisable. Therefore, explicit differentiation of the pseudopotential operators was avoided, only basis functions are differentiated.

30 $$\begin{aligned} H_{C}^{\lambda \eta }= &   \sum _{\mu \nu } P_{\mu \nu } \frac{\partial }{\partial \eta } \Big [(\delta _{\lambda A}-\delta _{\lambda C}) \langle \mu ^{(\lambda )}|U|\nu \rangle \nonumber \\  &   + (\delta _{\lambda B}-\delta _{\lambda C}) \langle \mu |U|\nu ^{(\lambda )}\rangle \Big ] \nonumber \\= &   2\sum _{\mu \nu } P_{\mu \nu } \Big [ (\delta _{\lambda A}-\delta _{\lambda C}) (\delta _{\eta A}-\delta _{\eta C}) \langle \mu ^{(\lambda \eta )}|U|\nu \rangle \nonumber \\  &   + (\delta _{\lambda A}-\delta _{\lambda C}) (\delta _{\eta B}-\delta _{\eta C}) \langle \mu ^{(\lambda )}|U|\nu ^{(\eta )}\rangle \Big ] \end{aligned}$$

The Kronecker delta in Eq. (30) tests only if $\lambda$ or $\eta$ correspond to the same atom where the differentiated atomic basis function is located. The derivative of a Cartesian Gaussian function is a combination of Gaussians with shifted angularity: ± 1 for first derivatives and ± 2 and 0 for second derivatives. Avoiding to differentiate the pseudopotential operator allows us to apply directly the semi-numerical integration algorithm developed for ECPs [45]. Evaluation of semi-local ECPs and local ECPs and MCPs’ second derivatives has a computational cost slightly larger than the evaluation of the corresponding energy integrals. The extra cost arises from the increased angularity of the required angular integrals which are, however, independent of the number of atoms. Non-local projectors in MCPs are easily decomposed into core-valence overlap integrals, and their derivatives can be evaluated very efficiently since they are completely analog to the overlap matrix derivatives.

**Table 1:** Analytic unrestricted ADFT harmonic frequencies (cm^–1^) of H_2_O calculated with the LDA VWN and GGA PBE functionals employing their native deMon2k and LibXC implementations. In the analytic frequency analyses, analytic kernel (ANK) and finite difference kernel (FDK) calculations are used. The H_2_O structure for these frequency analyses is depicted on the right. See text for further details

	

## Computational details

The open-shell analytical GGA exchange-correlation kernel formula, as detailed in the preceding section, Eq. (24), has been incorporated into the LCGTO-ADFT code deMon2k [46]. For the kernel calculations, either the native or LibXC partial derivatives of $e_{xc}(\tilde{\rho },\tilde{\gamma })$ are used. We denote them by native or LibXC in the following. The test calculations are performed with the Vosko, Wilk, and Nusair (VWN) LDA functional [47] in conjunction with Dirac exchange [48] or the GGA Perdew-Burke-Ernzerhof (PBE) functional [49] as implemented in deMon2k or LibXC. The Kohn-Sham orbitals are expanded with the double-zeta valence polarized (DZVP) basis set [50], while the auxiliary density is computed using the automatically generated GEN-A2 auxiliary function set [51]. Thus, the variational fitting of the Coulomb potential [5] is used, and the exchange-correlation energies, potentials, and kernels are calculated with the resulting auxiliary density. This ADFT approach is also used for the analytic frequency calculations. In all calculations, the self-consistent field (SCF) energy and auxiliary density convergence criteria are set to 10^-5^ a.u. and 5$\times$10^-4^ a.u., respectively. For the numerical integration of the exchange-correlation energy and its derivatives, an adaptive grid with a grid tolerance of 10^-5^ a.u. was used.

As benchmark calculations, we performed harmonic frequency analyses of (Pt_3_Cu)_n_ clusters with n being 1 to 11. The calculations are executed with the RPBE [52] GGA functional, again using either the native or LibXC implementation of it. In these calculations, the extended GEN-A2* auxiliary function sets [53] are used. For the platinum atoms, a double-zeta basis set from the Los Alamos National Laboratory was employed in conjunction with an 18-electron quasi-relativistic effective core potential (QECP| LANL2DZ) [54]. For the copper atoms, an all-electron triple-zeta valence polarized (TZVP) basis set optimized for GGA functionals [51] was used. Initial structures of the (Pt_3_Cu)$$ _{n}$$ clusters were taken from [11, 12] and tightly optimized using an SCF energy convergence criteria and an auxiliary density convergence criteria of 5$$\times $$10^-8^ a.u. and 10^-5^ a.u., respectively. The structure optimization tolerance was set to 3$$\times $$10^-4^ a.u. for the root-mean-square forces. The frequencies are calculated using the optimized cluster structures by analytic second ADFT energy derivatives employing ADPT. The required exchange-correlation kernels were calculated with the analytical and finite difference methods. In these calculations, a fixed fine grid was used for the numerical integration of the exchange-correlation energy, potential, and kernel. All calculations were performed using a 24-processor parallel architecture.

**Table 2:** Analytic unrestricted ADFT harmonic frequencies (cm^–1^) of small open-shell systems calculated with the LDA VWN and GGA PBE functionals employing their native deMon2k and LibXC implementations. In the analytic frequency analyses, analytic kernel (ANK) and finite difference kernel (FDK) calculations are used. The molecular structures are taken from [55]. See text for further details

Molecule	VWN				PBE			
	Native		LibXC		Native		LibXC	
	ANK	FDK	ANK	FDK	ANK	FDK	ANK	FDK
^2^BeH	1666.5	1666.0	1671.2	1672.9	1658.6	1659.0	1665.4	1664.8
^2^CH_3_ $\nu _1$	444.8	444.8	444.9	444.9	287.2	287.1	287.1	287.1
^2^CH_3_ $\nu _2$	1321.4	1321.3	1321.3	1321.1	1373.2	1373.0	1373.3	1373.0
^2^CH_3_ $\nu _3$	1321.4	1321.3	1321.3	1321.1	1373.2	1373.0	1373.3	1373.0
^2^CH_3_ $\nu _4$	3061.5	3061.5	3061.5	3061.5	3033.2	3033.2	3033.2	3033.2
^2^CH_3_ $\nu _5$	3237.1	3237.1	3237.2	3237.1	3201.3	3201.2	3201.3	3201.2
^2^CH_3_ $\nu _6$	3237.1	3237.1	3237.2	3237.1	3201.3	3201.2	3201.3	3201.2
^3^Li_2_	332.8	332.8	332.9	333.0	337.9	337.9	338.2	337.9
^3^Na_2_	162.1	162.1	162.1	162.2	166.3	166.2	166.2	166.2
^2^NH_2_ $\nu _1$	1452.0	1452.0	1451.9	1451.9	1493.4	1493.4	1493.4	1493.4
^2^NH_2_ $\nu _2$	3304.1	3304.1	3304.0	3304.0	3263.7	3263.7	3263.7	3263.7
^2^NH_2_ $\nu _3$	3407.5	3407.5	3407.3	3407.3	3369.0	3369.0	3368.9	3368.9
^3^NH	3190.0	3190.0	3189.7	3189.7	3149.2	3149.2	3149.2	3149.2
^2^NO	1990.2	1990.0	1990.2	1989.9	1997.5	1997.5	1997.5	1997.5
^3^O_2_	1524.4	1524.3	1524.4	1524.2	1536.5	1536.5	1536.5	1537.0
^2^PH_2_ $\nu _1$	1033.9	1029.0	1033.9	1029.6	1052.8	1052.5	1052.8	1052.6
^2^PH_2_ $\nu _2$	2321.9	2320.1	2321.7	2320.8	2346.5	2346.2	2346.4	2346.3
^2^PH_2_ $\nu _3$	2337.7	2334.3	2338.0	2335.6	2358.0	2357.7	2357.9	2357.9
^2^SiF	770.0	769.7	769.8	769.6	782.2	782.2	782.2	782.2
MAD	Ref.	0.6	0.4	0.8	Ref.	0.1	0.4	0.4

## Validation calculations

To validate the GGA ADFT kernel working equation, Eq. (24), we performed frequency analyses of the triplet H_2_O molecule at a not optimized geometry. To do so, we use the singlet VWN/DZVP/GEN-A2 optimized H_2_O structure, as depicted in Table 1, for our triplet frequency analyses. The obtained harmonic frequencies are listed in Table 1, too. Here, we compare analytic ADFT frequencies obtained with the LDA VWN functional and the GGA PBE functional. For both functionals, the native deMon2k functional implementation is compared with the corresponding LibXC implementation. For both implementations analytic (ANK) and finite difference (FDK), kernel results are listed. Each column displays the three harmonic frequencies of the triplet H_2_O at the optimized singlet geometry. As can be seen from Table 1, the first frequency is always imaginary (indicated by a minus sign). For the LDA VWN frequencies, excellent agreement between all methods is observed. Deviations are in the range of 1 cm^–1^ or below. The situation changes for the GGA PBE frequencies. Whereas the agreement of harmonic frequencies calculated with the analytic and finite difference kernels remains excellent, significant deviations between the native and LibXC kernel calculations are found. These differences are largest, up to 20 cm^–1^, for the imaginary frequency and reduce with increasing frequencies as Table 1 shows. We attribute this to different screenings in the native and LibXC partial derivatives of the density-weighted PBE exchange-correlation energy density. Similar deviations are found for other GGA functionals, too.

To further investigate the differences between the native and LibXC implementations of exchange-correlation functionals, we performed frequency analyses on small open-shell systems. In these unrestricted calculations, the VWN/DZVP/GEN-A2 and the PBE/DZVP/GEN-A2 level of theories were used. To be unbiased with respect to optimized structure parameters, we used structure parameters from the National Institute of Standards and Technology’s Computational Chemistry Comparison and Benchmark Database (NIST) [55] for the frequency analyses.

Table 2 compares the calculated harmonic frequencies of ten selected open-shell systems with each other. The table is structured in the same way as Table 1 adding mean absolute deviations (MADs) with respect to the native deMon2k ANK results at the bottom of the table. As Table 2 shows, a general good to excellent agreement between all frequencies is observed. This is confirmed by the MADs that are always below 1 cm^–1^. In particular, the difference between harmonic frequencies from the native and LibXC functional implementations is reduced well below 10 cm^–1^. Largest deviations are observed for ^2^BeH, both for the LDA VWN ($\sim$ 5 cm^–1^) and GGA PBE ($\sim$ 7 cm^–1^) functionals. Furthermore, Table 2 shows that the differences between native and LibXC implementations are now in the same size range as for analytic (ANK) and finite difference (FDK) kernel calculations ($\sim$ 5 cm^–1^ for ^2^PH_2_ $\nu _1$ with the LDA VWN functional). This supports our previous assumption that these differences arise from different screenings in the functional implementations. We attribute the improvement of the consistency of the frequencies in Table 2 with respect to Table 1 to the molecular geometries. Whereas the molecular structure parameters for the systems in Table 2 are close to optimized structure data, the triplet H_2_O structure used in Table 1 is far from the corresponding minimum. This is also supported by the fact that the largest deviation between native and LibXC functional implementations is observed for the imaginary frequency of the triplet H_2_O example.

**Table 3:** Computational timings (s) for finite difference (FD) and analytic (AN) ECP second derivative calculations of (Pt_3_Cu)_n_ clusters, with *n*=1 to 11. The speed-up factor refers to frequency analyses performed on 24 cores

Cluster	FD	AN	Speed-up
^2^Pt_3_Cu	2.2	0.2	11.5
^3^Pt_6_Cu_2_	13.8	0.7	20.0
^4^Pt_9_Cu_3_	43.4	1.5	28.8
^3^Pt_12_Cu_4_	102.8	2.8	36.3
^4^Pt_15_Cu_5_	182.8	4.1	44.4
^3^Pt_18_Cu_6_	295.2	5.4	54.8
^2^Pt_21_Cu_7_	504.7	8.2	61.8
^5^Pt_24_Cu_8_	756.5	10.8	69.9
^2^Pt_27_Cu_9_	1064.3	13.6	78.5
^3^Pt_30_Cu_10_	1594.2	18.3	87.1
^6^Pt_33_Cu_11_	2202.2	23.5	93.5

**Table 4:** Analytic unrestricted ADFT harmonic frequencies (cm^–1^) of (Pt_3_Cu)_n_ clusters, with *n*=1 to 11, calculated with the GGA RPBE functional using its native deMon2k and LibXC implementations. In the analytic frequency analyses, analytic kernel (ANK) and finite difference kernel (FDK) calculations are used. The molecular structures are re-optimizations of the structures reported in [11, 12]. See text for further details

Native										
Cluster	ANK					FDK				
	$\nu _1$	$\nu _2$	$\nu _3$	$\nu _4$	$\nu _5$	$\nu _1$	$\nu _2$	$\nu _3$	$\nu _4$	$\nu _5$
^2^Pt$$ _{3}$$Cu	89.4	89.5	136.3	137.5	177.5	89.4	89.5	136.3	137.5	177.5
^3^Pt$$ _{6}$$Cu$$ _{2}$$	14.2	36.6	41.6	61.6	75.3	14.2	36.6	41.6	61.6	75.3
^4^Pt$$ _{9}$$Cu$$ _{3}$$	41.1	46.1	57.1	61.9	63.4	41.1	46.0	57.0	62.0	63.4
^3^Pt$$ _{12}$$Cu$$ _{4}$$	16.1	37.5	37.9	47.1	47.8	16.2	37.5	37.9	47.1	47.8
^4^Pt$$ _{15}$$Cu$$ _{5}$$	34.9	39.9	44.8	49.3	51.3	34.9	39.7	44.5	49.1	51.0
^3^Pt$$ _{18}$$Cu$$ _{6}$$	23.7	36.0	40.4	42.7	47.8	23.3	36.2	40.3	42.8	47.9
^2^Pt$$ _{21}$$Cu$$ _{7}$$	28.8	31.8	34.6	35.9	43.0	28.0	31.0	34.3	35.3	42.7
^5^Pt$$ _{24}$$Cu$$ _{8}$$	23.9	29.9	34.5	35.9	39.4	24.4	30.6	34.2	36.4	39.8
^2^Pt$$ _{27}$$Cu$$ _{9}$$	6.8	15.1	16.6	25.6	29.0	8.7	15.9	19.3	25.5	29.2
^3^Pt$$ _{30}$$Cu$$ _{10}$$	28.0	28.8	34.6	35.9	37.9	28.1	29.3	34.3	36.2	37.1
^6^Pt$$ _{33}$$Cu$$ _{11}$$	28.3	32.5	33.8	34.7	37.8	29.3	32.8	34.0	34.4	37.8
MAD	Ref.	Ref.	Ref.	Ref.	Ref.	0.4	0.3	0.4	0.2	0.2
LibXC										
Cluster	ANK					FDK				
	$\nu _1$	$\nu _2$	$\nu _3$	$\nu _4$	$\nu _5$	$\nu _1$	$\nu _2$	$\nu _3$	$\nu _4$	$\nu _5$
^2^Pt$$ _{3}$$Cu	88.3	89.7	136.3	137.5	177.8	88.2	89.7	136.2	137.5	177.7
^3^Pt$$ _{6}$$Cu$$ _{2}$$	18.1	36.2	44.4	61.9	75.1	19.1	36.4	44.5	61.4	74.9
^4^Pt$$ _{9}$$Cu$$ _{3}$$	46.4	46.9	57.8	62.6	65.9	47.5	56.1	62.2	64.0	68.8
^3^Pt$$ _{12}$$Cu$$ _{4}$$	14.1	37.3	40.4	46.6	48.7	12.9	36.5	39.9	45.8	48.2
^4^Pt$$ _{15}$$Cu$$ _{5}$$	33.8	33.9	44.4	48.8	50.2	33.9	40.7	44.6	49.4	51.8
^3^Pt$$ _{18}$$Cu$$ _{6}$$	22.7	36.3	40.7	43.2	47.7	22.2	36.1	40.1	43.1	47.6
^2^Pt$$ _{21}$$Cu$$ _{7}$$	29.7	33.6	35.0	36.1	44.3	29.7	33.6	35.0	36.1	42.9
^5^Pt$$ _{24}$$Cu$$ _{8}$$	27.1	29.7	32.4	36.7	39.6	27.0	30.6	32.5	36.5	39.0
^2^Pt$$ _{27}$$Cu$$ _{9}$$	2.6	6.9	18.1	27.3	28.7	6.0	7.2	14.3	26.3	27.5
^3^Pt$$ _{30}$$Cu$$ _{10}$$	27.9	28.6	34.2	36.2	38.2	27.9	28.6	34.2	36.2	38.2
^6^Pt$$ _{33}$$Cu$$ _{11}$$	31.9	33.5	35.1	38.1	41.4	31.7	34.4	34.4	38.4	40.2
MAD	2.4	1.8	1.1	0.8	1.0	2.4	2.3	1.5	0.9	1.3

## Benchmark calculations

To benchmark our new GGA ADFT kernel implementation, we revisited the (Pt_3_Cu)_n_ clusters previously reported in [11, 12]. To this end, we re-optimized the cluster structures and performed frequency analyses with the optimized structures employing the native and LibXC implementations of the RPBE [52] functional. We used this functional because of its use in the original report. Due to the computational demand of these calculations, we profiled some of them. To our surprise, we found that a significant portion of the computational time was spent on the finite difference calculation of the ECPs. Table 3 lists these timings in column 2.

**Fig. 1:**
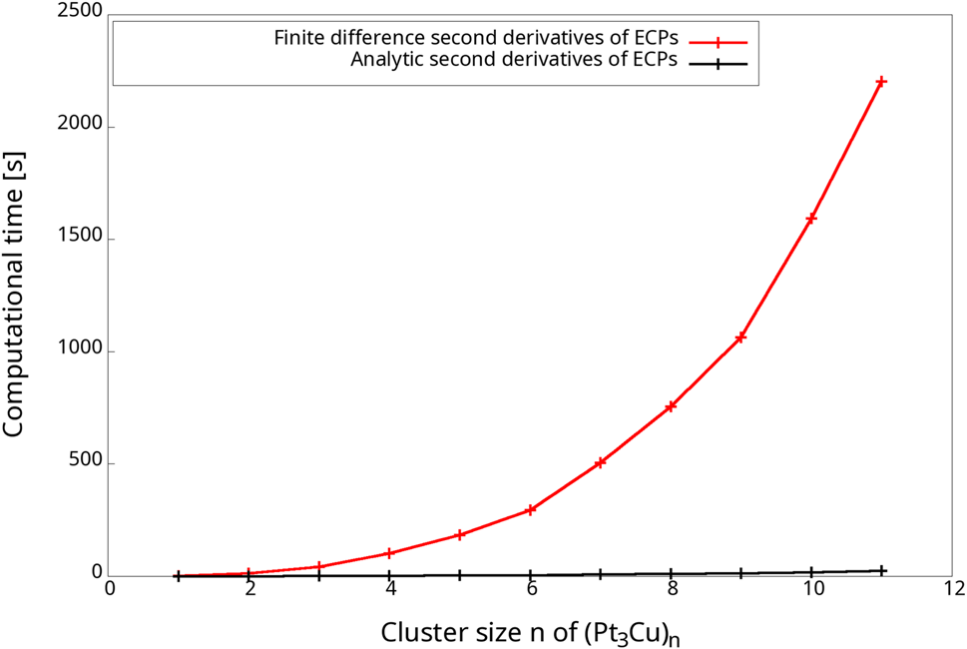
Comparison of the computational timings for finite difference and analytic ECP second derivative calculation of (Pt_3_Cu)_n_ clusters, with *n*=1 to 11. All calculations are performed on 24 cores

To overcome this computational bottleneck analytic second ECP (and MCP), derivatives were implemented in deMon2k as described in Section 2.4. The timings for the analytic ECP second derivative calculations are reported in column 3 of Table 3 and graphically compared with their finite difference counterparts in Fig. 1. Speed-ups of up to almost 100 are observed for the studied (Pt_3_Cu)_n_ clusters! As Fig. 1 shows, the newly analytic implementation of ECP second derivatives exhibits a nearly linear scaling with respect to the number of ECP centers. Note that the semi-local integrator scaling is only due to the numerical radial integrator [45] because analytic angular integration is independent of the number of pseudopotential centers in the system.

The five lowest harmonic frequencies of the optimized (Pt_3_Cu)_n_ are listed in Table 4 for the native and LibXC functional implementations. The MADs with respect to the native deMon2k ANK frequencies are given at the end of the table. Again, the analytic (ANK) and finite difference (FDK) kernel calculations are compared for both implementations. Notably, there is an outstanding agreement between the analytic and finite difference kernel calculations as shown by the corresponding MADs that are all below 0.5 cm^–1^. Deviations rarely exceed three wavenumbers, underscoring the robustness of the deMon2k implementation. In contrast, the results obtained with the LibXC interface exhibit more deviations. While deviations are generally less than two wavenumbers (maximum MAD is 2.4 cm^–1^), some frequencies display discrepancies of up to 10 wavenumbers. Additionally, using the LibXC interface with the above-mentioned methodology, two systems revealed imaginary frequencies in the case of the FDK method, namely Pt_6_Cu_2_ and Pt_27_Cu_9_. To overcome this problem, we re-optimized these two clusters with a tighter structure optimization tolerance of 10^-5^ a.u. and an analytically calculated start Hessian matrix. In the subsequent frequency analyses, only positive frequencies are observed. For consistency, both ANK and FDK frequencies for Pt_6_Cu_2_ and Pt_27_Cu_9_ listed in Table 4 were obtained with this methodology. These results show that the here-described ADFT kernel implementations are numerically stable for delicate electronic structures typical of transition metal clusters.

## Conclusions

The unrestricted GGA ADFT exchange-correlation kernel working equation for perturbation dependent auxiliary functions is derived and discussed. We validated and benchmarked this kernel implementation in the framework of frequency analyses using the native and LibXC exchange-correlation functional implementations in deMon2k. Overall, there is a very satisfying consistency between the native and LibXC frequencies. In both cases, deviations of less than a wavenumber are typically observed when comparing analytic and finite difference kernel calculations. Between the implementations, larger deviations of 5 cm^–1^ or more are occasionally observed. The largest deviation ($\sim$ 20 cm^–1^) between native and LibXC frequencies is found for the imaginary mode in the non-minimum structure of ^3^H_2_O. We attribute this to the different screenings in the exchange-correlation energy density calculations. However, for (near) minimum structures, the agreement of native and LibXC frequencies is for small open-shell molecules usually excellent. In the case of (Pt_3_Cu)_n_ clusters, results obtained using the LibXC interface show more significant deviations, albeit generally within 2 wavenumbers, with occasional discrepancies of up to 10 wavenumbers. In the case of Pt_6_Cu_2_ and Pt_27_Cu_9_, these deviations led to imaginary frequencies in the final optimized structures with the default convergence thresholds. By tightening these thresholds and using an analytically calculated start Hessian matrix, these problems were fixed and the clusters converged with the LibXC functional implementation to minima, too. The computational bottleneck due to the finite difference second derivative ECP (and MCP) integral calculations in the (Pt_3_Cu)_n_ clusters was resolved by the implementation of corresponding analytic second derivatives. The resulting speed-ups are large and scale linearly with the number of ECP (MCP) centers. Consequently, frequency analyses of systems with hundreds of ECP (MCP) centers are now feasible with deMon2k in very reasonable times.

## Data Availability

No datasets were generated or analyzed during the current study.
